# Grain Orientation Optimization of Two-Dimensional Grain Selector during Directional Solidification of Ni-Based Superalloys

**DOI:** 10.3390/ma13051121

**Published:** 2020-03-03

**Authors:** Xintao Zhu, Qiang Yang, Fu Wang, Dexin Ma

**Affiliations:** 1 State Key Laboratory for Manufacturing System Engineering, School of Mechanical Engineering, Xi’an Jiaotong University, Xi’an 710049, Shanxi, China; zhudb8@gmail.com (X.Z.); yangqiang@stu.xjtu.edu.cn (Q.Y.); 2 Foundry Institute, RWTH Aachen University, Intzestrasse 5, 52072 Aachen, Germany; d.ma@gi.rwth-aachen.de

**Keywords:** 2D grain selector, single crystal, directional solidification, take-off angle, deviation of the grain orientation

## Abstract

The grain selection method is widely used in industry to produce Ni-based single crystal superalloys. A Z-form two-dimensional (2D) grain selector was designed to obtain high-quality single crystals. To control grain orientation deviation, one of the most important defects of the single crystal superalloys in casting, Z-form 2D grain selectors with different take-off angle were investigated in this study. The MM247LC superalloy single crystal samples were obtained by the Bridgman method modified by the Z-form grain selectors in this study. The Electron Backscattered Diffraction (EBSD) and the Optical Microscopy (OM) were used to observe and measure the grain selection growth and the microstructural evolution and orientation of the single crystal were also discussed. The results show that a Z-form 2D grain selector with an appropriate take-off angle can significantly reduce the deviation of the grain orientation. A single crystal superalloy with a deviation angle less than 6° can be obtained effectively when the take-off angle was 40°.

## 1. Introduction

Jet engine turbine blades are located in the harsh environment behind the burner and rotate at high speed to extract energy from the high-temperature gas stream. Generally speaking, single-crystal Ni-based superalloy is usually used in the gas turbine to optimize its functions and to perform the roles of energy conservation, emission reduction and environmental protection [1,2,3]. To obtain an excellent performance of superalloys, a lot of research has been carried out. Wretland and Polvorosa proposed a method to improve the properties of alloy by refining grain [4,5]. After decades of development, the directional solidification (DS) casting technology of alloys has been greatly advanced. Nowadays, Ni-based single crystal (SX) superalloy turbine blades have been widely used in jet engines and power generation for their endurance under high service temperature, their thermomechanical fatigue properties, and their creep performance [6,7,8,9,10,11]. The primary aims of producing SXs are to eliminate grain boundaries that limit creep ductility and to orient the elastically soft <001> orientation parallel to the maximum load to minimize cyclic stresses during thermal cycling [12]. The transition from Directional Solidification to Single Crystal Casting was achieved by introducing a grain selector consisting of two parts, a grain orientation-optimized starter block and a selector part [13,14,15].

To obtain a single crystal structure with superior mechanical properties, the design of the grain selector is a crucial factor in the casting process. Several kinds of grain selectors, such as restrictor, angled, and spiral selectors, have been designed in the past few years in order to develop a high-efficiency grain selector for growing SX blades [16]. However, the application of restrictor selector has been limited due its lower degree efficiency, resulting a longer length for selecting a single grain [17]. Around the corners of the angled selector, new grains often nucleate due to the sudden change in growth direction [18,19]. In the wax injection stage, the spiral selector cannot be integrally fabricated with the blades, because of the complex, three-dimensional (3D) shape of the spiral. It needs to be welded with the blades, inevitably resulting in some position error and sudden failure in the grain selection. The high stability of the spiral selector cannot be maintained, although it has been employed in casting foundries.

To overcome the shortcomings of traditional selectors, the 2D grain selector was proposed as a candidate to obtain a high-quality single crystal. Esaka developed a mathematical model for a 2D grain selector [20]. Moreover, Gheisari and Karamian studied the dendritic growth mechanism in a 2D Zigzag grain selector [21]. A C-form and Z-form 2D grain selector were also put forward, and the effect of the geometry of the selector on grain selection was investigated by our research group in previous works [1,22]. In this paper, a Z-form 2D grain selector was designed to obtain high-quality single crystals, and the effect of the take-off angle of 2D selectors on grain selection and orientation was further investigated, and the take-off angle of Z-form 2D selectors was optimized in this paper to obtain high single crystal selection efficiency and a lower deviation angle.

## 2. Experiments

### 2.1. Mode of Z-form 2D Grain Selector

**Figure 1:**
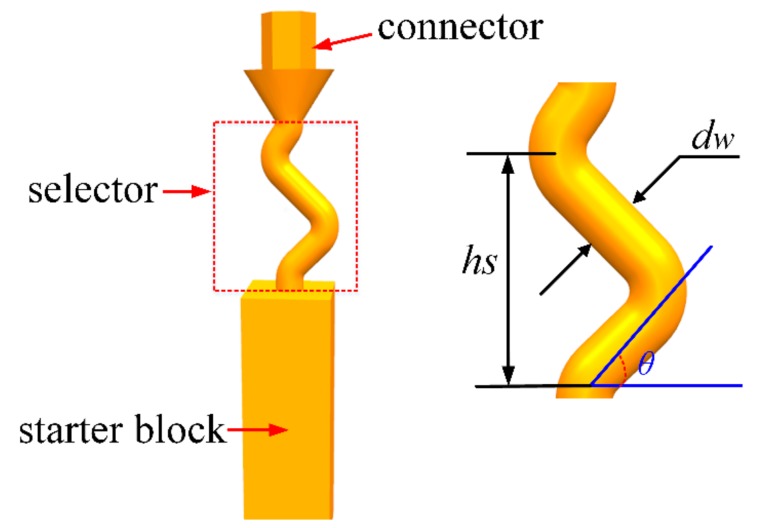
Schematic drawings of the Z-form 2D grain selector and its key geometric parameters.

The Z-form 2D selector is mainly divided into three plates: starter block, z-selector and connector, as shown in Figure 1. In this study, the starter block with a polygon combination of 10 mm × 10 mm × 30 mm, the diameter of grain selector (*dw*) of 3 mm and the height (*hs*) of 30 mm are fixed. The take-off angle (*θ*), one of the most important geometrical parameters of the Z-form 2D selector, was varied between 15°, 30°, 40°, 50°, and 60°, as shown in Table 1, to find the best parameter for grain selection and an optimization grain orientation.

### 2.2. Materials and Experiment Procedures

In this paper, the Ni-based superalloy of MM247LC (Doncasters Group Ltd., Repton, UK) was used as raw materials, and the chemical composition of the superalloy is shown in Table 2. First of all, the wax pattern of Z-form 2D grain selectors with different take-off angles were fabricated by a 3D printer (P4K 75, ENVISIONTEC GMBH, Gladbeck, Germany). The second step was to put the wax patterns into the ceramic slurry and sand them with alumina of different sizes and lengths. Circular dip coating was not completed until the thickness reached 7~8 mm. After drying, the mold patterns were dewaxed, and the mold was further sintered to remove the remaining wax and reinforce the strength of the shell. Finally, the mold could be installed on the water-cooled copper cooler of ALD vacuum furnace (ALD Vacuum Technologies GmbH, Hanau, Germany). In the process of investment casting, the shell mold was raised to a cylindrical heater and heated to 1470 °C. After equalizing the furnace temperature, the melted alloy was heated to 1500 °C and poured into the mold. The mold was withdrawn from the furnace with extraction rates of 3 mm/min. Once the heater temperature dropped below 300 °C, the vacuum was released and the mold was removed. Finally, the mold was knocked out, and the units and cluster were separated.

### 2.3. Characterization

After removing the ceramic debris, the selectors were etched to reveal the macrostructure using a 50% H_2_O_2_ + 50% HCl etchant. The selectors were then cut longitudinally and transversely and etched using a 60 mL C_2_H_5_OH + 40 mL HCl + 2g (CuCl_2_∙2H_2_O) etchant to show the dendrite structure in the selector. The structure of dendrite is relatively complex, the optical microscope (OM, ZEISS, Oberkochen, Germany) and the scanning electron microscope and EBSD (SEM, Hitachi SU3500, Tokyo, Japan) was used to study the evolution trend and dynamic change of dendrite structure.

## 3. Results and Discussion

**Figure 2:**
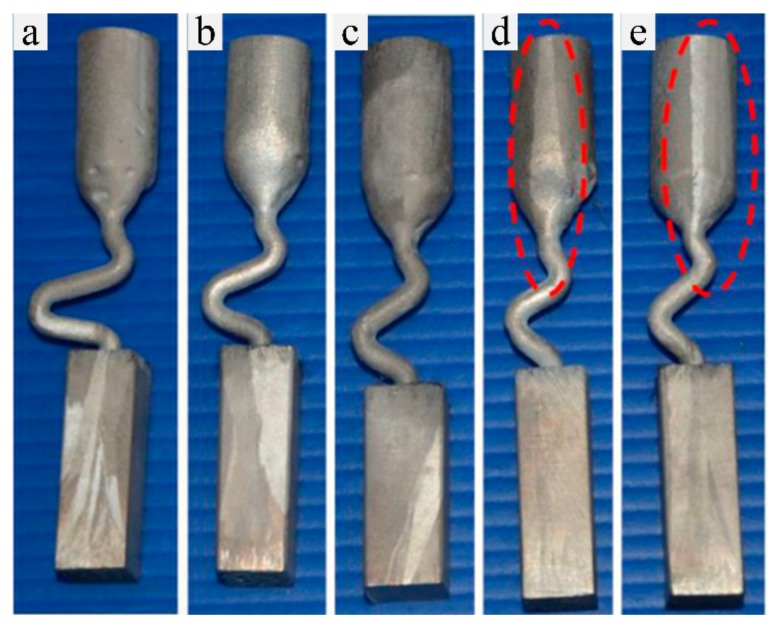
Z-form 2D grain selector with different take-off angle: (**a**) θ = 15°, (**b**) θ = 30°, (**c**) θ = 40°, (**d**) θ = 50°, (**e**) θ = 60°.

Five Ni-based superalloys samples fabricated by Bridgeman directional solidification process with different take-off angles (θ) were obtained, as shown in in Figure 2. The results of single crystal analysis are shown in Table 3.

As can be seen in Figure 2 and Table 3, a single crystal microstructure could only be achieved in Case 1, Case 2 and Case 3 with the take-off angle of 15°, 30° and 40°, respectively, while stray grains exist in the grain selectors with take-off angle larger than 40°. Therefore, it can be seen that the take-off angle of 2D grain selector has a direct effect on the single crystal growth.

**Figure 3:**
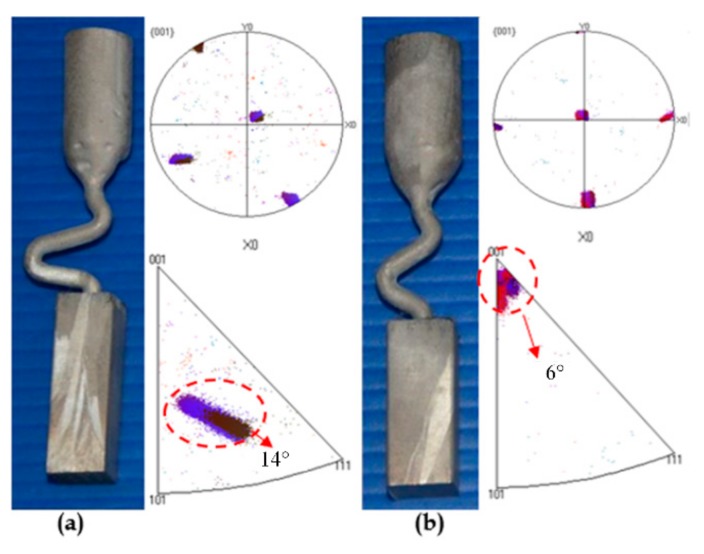
Pole figures of two cases with different take-off angles: (**a**) θ = 15°; (**b**) θ = 40°. The take-off angle also influences the deflection angle to <001> orientation.

Anisotropy of grain is a fundamental feature of single crystals. Due to the cubic crystal structure of the Ni-based high-temperature alloy, it has a minimum modulus of elasticity in the <001> direction. The stress required to deform the high-temperature alloy along the <001> direction is minimal. The mechanical properties of Ni-based superalloys are mainly determined by the final orientation. Controlling the <001> direction is a key point in optimizing the grain selection. To further investigate the effect of take-off angle on the quality of single crystal, EBSD analysis on the selected single crystal was carried out to study the effect of take-off angle on the deviation of the grain orientation. As for selector Case1 and Case3, the microstructures of the chosen cross-sections were single grain microstructures. For every sample, 3~6 points were scanned by EBSD, but only one representative map was chosen to show the deviation angle of gain. For selector Case1, the deviation angle was about 14° while for Case 3, the deviation angle was about 6°, as shown in Figure 3. It can be seen that the take-off angle also influences the deviation angle in the <001> orientation.

As shown in Figure 2 and Figure 3, the reason for the results can be speculated. For grain selectors with a small take-off angle like in Figure 3a, when dendrites grow into the Z-form grain selector part, growth of most dendrites is limited by the wall, except for the grains close to Z-form grain selection side. Therefore, the single grain structure could be achieved by a grain selector with a small take-off angle. However, because the surviving dendrites are from the outward part instead of the core of the wire which connects the starter block and the grain selector part, the deviation angle of the surviving dendrites is usually large. It can explain why the final deviation angle of grain selector with small take-off angle (θ = 15°) is as large as about 14°. In terms of the grain selector with a take-off angle as shown in Figure 3b, there would be a larger chance that the dendrites from the outward part as well as the core of the connection wire could survive. Usually the dendrites from core of the connection tube would grow into the final main grains, while the outward grains are stray grains. As a result of that, the selector with large take-off angle would have a small deviation angle, while having a smaller chance to achieve single grain structure.

**Figure 4:**
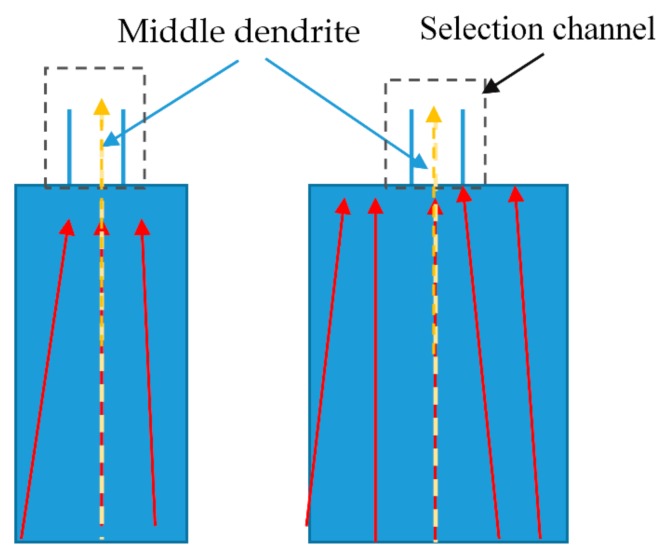
Grain growth direction in the start block.

Figure 4 shows that the intermediate yellow dendrites have a better <001> orientation. The dendrites participating in the competitive growth in selector channel determine the orientation of the final blade. Previous articles by Zhu et al. showed that the starter block with a height between 10 mm and 30 mm can optimize the top <001> orientation within 10 degrees, which can meet the requirements of industrial production [1,22].

To better study the influence of the position of the top grain selection channel on the final orientation, we controlled the angle of the take-off angle of the selected section to 15°, 30°, 40°, 50° and 60°, the selector diameter was fixed at an optimized value 3 mm. First, the angle of dendrite competition in the selector part was investigated as shown below.

**Figure 5:**
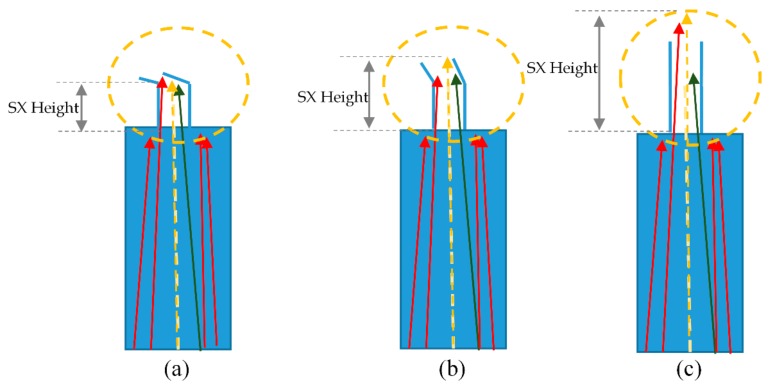
Grain growth direction in selector part with different take-off angles. (**a**) θ < 45°; (**b**) θ = 45°; (**c**) θ > 45°.

Figure 5a shows that when the take-off angle gets smaller (less than 45°), the red dendrite on the left can win in the fast competition, and the middle yellow dendrite and the right green dendrite are blocked. It can be seen from the model that the smaller the take-off angle, the more easily the red dendrites are selected. However, the red dendrites are not oriented as <001> in the middle of the yellow dendrites. The smaller the take-off angle, the lower the final selected single crystal height is. However, the final <001> orientation cannot be optimized. From Figure 5b, we can see that when the take-off angle is greater than or equal to 45°, the yellow dendrites can effectively compete among the three dendrites. Because the yellow dendrites are better at <001> orientation, they can grow faster. The initial take-off angle can be controlled to about 45° so that the yellow dendrites can be selected from other dendrites with poor <001> orientation. As can be seen from Figure 5c, when the take-off angle is close to 90°, the final yellow dendrite has a higher probability of winning in terms of competitive growth, and the final orientation <001> is also the best. However, the grain selection height is increased a lot, and the grain selection efficiency is poor. Moreover, it is easy to form stray grain. The starting angle can be optimized at about 45°, so that the middle dendrite can win in the selection with efficient <001> orientation and sufficient grain selection efficiency. Then, the difference between the critical take-off angle of 45° and the upper and lower dendrites will be discussed.

**Figure 6:**
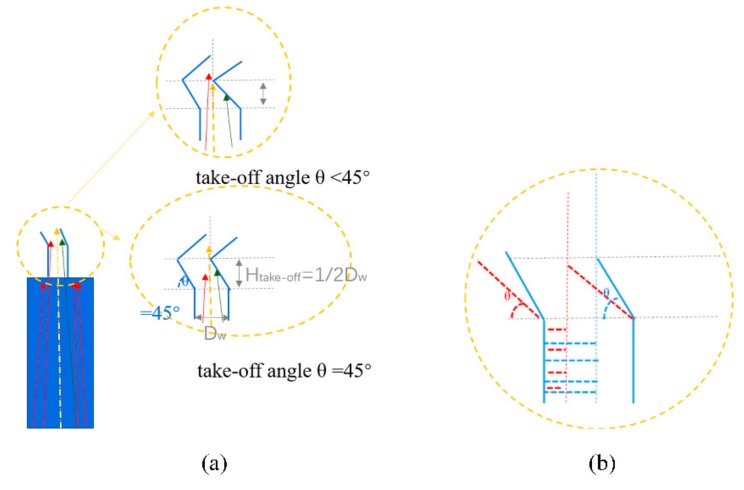
(**a**) The upper and lower dendrites at the critical take-off angle of 45° and (**b**) the comparison of red and blue dendrites growth channel with related take-off angle changing.

**Figure 7:**
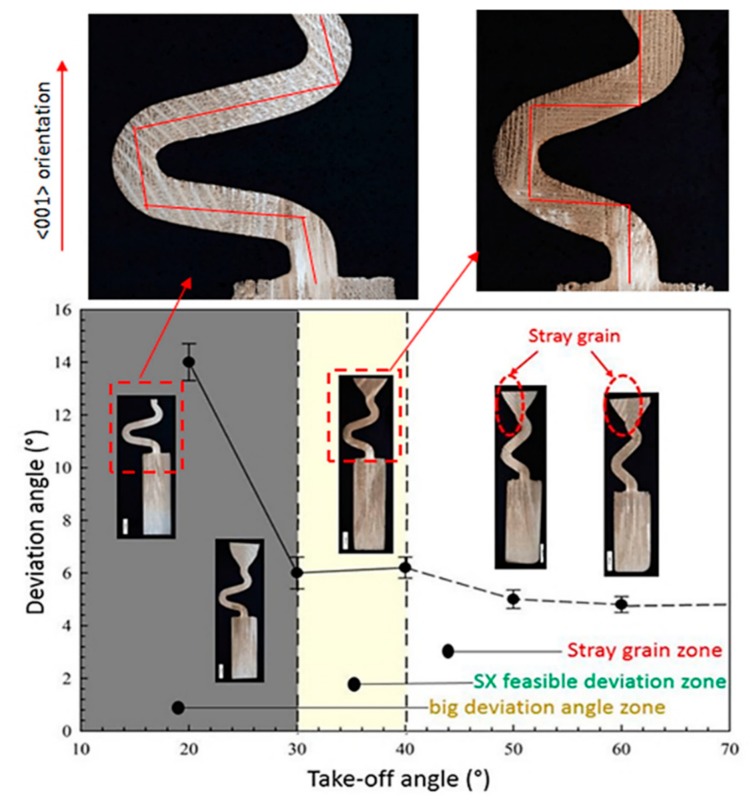
Correlation between deviation angle and take-off angle.

From Figure 6a, we can see that when the take-off angle is less than 45°, the red dendrite <001> orientation on the left is more likely to preferentially pass through the corner passage. The middle dendrite is blocked because when the H_take-off_ is less than 1/2D_w_, yellow dendrites cannot pass. Figure 6b shows that when the take-off angle gets smaller, the selector has a larger take-off angle, as indicated by the blue dotted line, allowing more dendrites to pass through the corner. In this model, the smaller the take-off angle, the smaller the red area, meaning that less dendrites pass near the left edge, which leads to the inability of the better-oriented dendrites in the middle. Although this can improve the efficiency of grain selection, the final orientation is not optimized. As a result, selectors with large take-off angles would have a small deflection angle and a smaller chance of achieving a single grain structure. This analysis can be confirmed in Figure 7.

**Figure 8:**
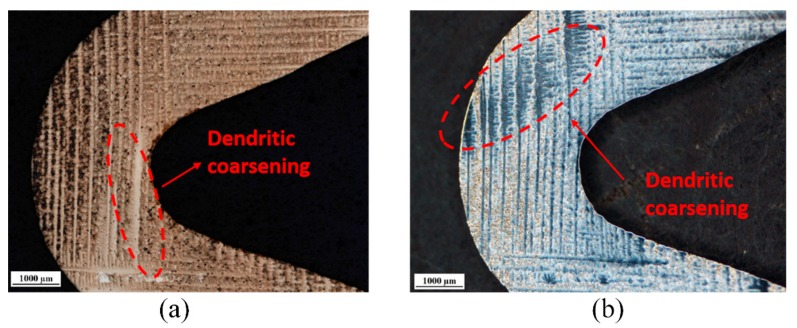
The dendrite coarsening occurring in the first corner (**a**) and the second corner (**b**) of the selector part.

The best experimental 40° angle of the final experimental results of MM247LC was metallographic analyzed by cutting from the middle of the longitudinal plane. Figure 8a shows that, after corrosion, the coarse phenomenon of dendrites in the <001> direction appears at the turns, and the dendrites are likely to appear on the inner side of the turns. Figure 8b shows that, after re-grinding the 0.5 mm metallographic phase in the longitudinal section, the lateral dendrite at the second bend of the Z-form appeared on the outside of the turn. Dendritic segregation in directional solidification mainly depends on the solute distribution coefficient and the homogenization effect of diffusion. The change in elemental segregation depends mainly on the homogenization effect of the diffusion, while the latter mainly depends on the diffusion time and the spreading distance. This experiment was carried out at a balanced withdraw rate of 3 mm/min.

**Figure 9:**
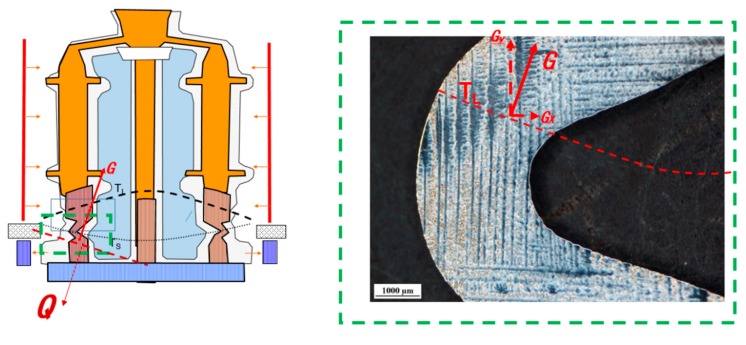
The 2D dendrite morphologies of overhang regions integrated with thermal profile for MM247LC.

It can be seen that the predicted local isotherms on the turning area can break into Gx and Gy. By correlating the thermal profile and the dendrite growth path, the dendrite structure evolution can be determined. At the initial stage, the secondary arms of primary dendrites within the main body were extended laterally into the lower overhang region and reached the extremity. The secondary dendrites grew much faster than the original primary ones due to a high tip undercooling. When the secondary dendrites were extended, the ternary dendrites propagated along the solidification direction. Finally, the turning region was filled by the ternary dendrite array. The Gx contributes to the dendritic coarsening, as shown in Figure 9.

**Figure 10:**
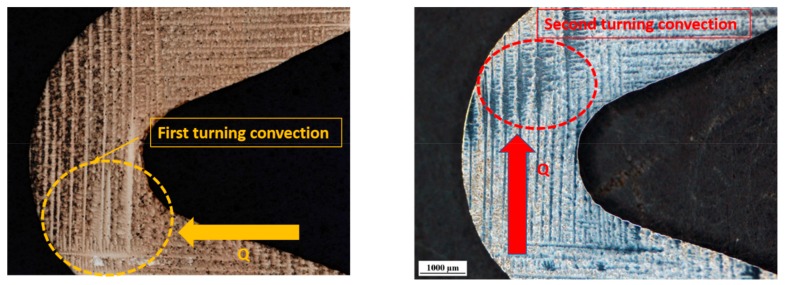
The dendrite turning convection relationship with the heat flow.

Figure 10 indicates that the dendrite coarsening always happened in the turning area and had a nearly 90° angle with the heat flow regularity Q, based on the experimental results. According to the schematic diagram shown in Figure 9 and Figure 10, at the first turn of the Z-form, the lateral dendrites and the longitudinal dendrites grow simultaneously, while the longitudinal dendrites have priority in the heat flow and the <001> direction, and the inner longitudinal dendrites preferentially grow, and are thus inhibited. The diffusion of the lateral dendrites on the left side causes the longitudinal dendrites to coarsen. The lateral dendrites on the left cannot diffuse, resulting in a vertical growth environment, which causes the vertical dendrites on the left side of the competition segment to be coarsened. From the perspective of the dendritic competition geometric barrier, the competition dendrite coarsening inhibited the growth of the lateral dendrites on the right side, and played a positive role in the longitudinal dendrites blocking the lateral dendrites. The dendrite coarsening caused by uneven concentration distribution during dendritic competition and growth has a positive blocking effect on grain selection. It can also be seen that dendrite coarsening also occurs at the second turn of the Z-form grain selector near the outer side. From the metallographic diagram, the coarsening phenomenon is mainly concentrated on the lateral dendrites, while the inner side of the second turn is not prone to coarsening.

## 4. Conclusions

The effect of take-off angle, a key geometrical parameter of Z-form 2D grain selectors, on grain selection and the orientation was investigated experimentally by using optical microscopy and EBSD techniques. The results obviously showed that the Z-form grain selector exerted a great effect on grain orientation during the Ni-based single crystal turbine blades casting by directional solidification, and the take-off angle of Z-form 2D grain selector had a significant effect on grain selection and orientation. The Z-form 2D grain selector with a 40° take-off angle (θ) is recommended as the optimal angle, with a high single crystal selection efficiency and a lower deviation angle of 6°. The phenomenon of turning dendrite coarsening was mainly caused by the rapid change of the heat flow vector at the turn, which leads the solute to diffuse slowly and accumulate at the turns, resulting in a decrease in the cooling rate of solidification.

## Figures and Tables

**Table 1:** The designed parameters of the take-off angle of different grain selector.

Case	1	2	3	4	5
θ/deg	15°	30°	40°	50°	60°
d_w_/mm	3	3	3	3	3

**Table 2:** The chemical composition of superalloy MM247LC (wt. %).

Elements	Al	Ti	Cr	Mo	Co	W	Ta	Hf	C	Ni
Wt.%	5.49	0.74	8.03	0.5	9.41	9.87	2.9	1.36	0.094	Bal.

**Table 3:** Grain selection of Z-form grain selector with different take-off angles.

Case	1	2	3	4	5
θ/deg	15°	30°	40°	50°	60°
Single Crystal	Yes	Yes	Yes	No	No
